# Whole-Genome Sequences of Clinical Enterobacter bugandensis Isolates from Germany

**DOI:** 10.1128/MRA.00465-19

**Published:** 2019-07-18

**Authors:** Jane Falgenhauer, Can Imirzalioglu, Linda Falgenhauer, Yancheng Yao, Anja M. Hauri, Beate Erath, Oliver Schwengers, Alexander Goesmann, Harald Seifert, Trinad Chakraborty, Swapnil Doijad

**Affiliations:** a Institute of Medical Microbiology, Justus-Liebig-University Giessen, Giessen, Germany; b German Center for Infection Research (Partner Site Giessen-Marburg-Langen), Giessen, Germany; c Hesse State Health Office, Centre for Health Protection, Dillenburg, Germany; d Klinikum Bad Hersfeld GmbH, Bad Hersfeld, Germany; e Bioinformatics and Systems Biology, Justus-Liebig-University Giessen, Giessen, Germany; f Institute for Medical Microbiology, Immunology and Hygiene, University Hospital of Cologne, Cologne, Germany; g German Center for Infection Research (Partner Site Bonn-Cologne), Bonn, Germany; University of Maryland School of Medicine

## Abstract

Enterobacter bugandensis is the most pathogenic species of the genus *Enterobacter* and is a cause of life-threatening infections in neonates. Curiously, it was also detected in samples from the International Space Station. Here, we present complete closed genome sequences of two clinical *E. bugandensis* isolates recognized for the first time in Germany.

## ANNOUNCEMENT

Infections with *Enterobacter* spp. are notoriously difficult to treat, and broad resistance to third-generation cephalosporins and quinolones is an increasing problem (1). *Enterobacter* species are members of the ESKAPE (Enterococcus faecium, Staphylococcus aureus, Klebsiella pneumoniae, Acinetobacter baumannii, Pseudomonas aeruginosa, and *Enterobacter* species) group of pathogens and are on the WHO priority list for developing new antimicrobials (2). Members of this genus are ubiquitous in nature, but some species are opportunistic human pathogens, particularly those causing neonatal infections and infections associated with the urinary tract, respiratory tract, and bloodstream. Recently, a novel species, Enterobacter bugandensis, was identified from a neonatal outbreak in Tanzania with a 35% case fatality rate, and it is currently considered the most pathogenic member of this genus (1, 3). Since the original description, *E. bugandensis* was only reported from the International Space Station as a multidrug-resistant species persisting under microgravity conditions (4). Here, we report the first recognition of *E. bugandensis* involved in clinical cases in Germany.

Two isolates, F-1367 and Survcare220, were recovered on 5% sheep blood agar from the blood culture of a neonatal infection and the throat swab of an 83-year-old patient, respectively. Routine species identification using matrix-assisted laser desorption ionization–time of flight (MALDI-TOF) mass spectrometry (MS) (Vitek) and the Vitek 2 Gram-negative (GN) identification (ID) card (bioMérieux, France) classified them as *Enterobacter* spp. but did not permit species allocation.

For both isolates, complete genome sequences were generated by combining long reads from Oxford Nanopore Technology (ONT) and short reads from Illumina technology. In brief, DNA was isolated from overnight cultures in LB broth using a PureLink genomic DNA kit (Thermo Fisher, Germany). For long reads, libraries were prepared with a native barcoding kit (EXP-NBD103) and 1D chemistry (SQK-LSK108). Sequencing was carried out on a MinION instrument, using a SpotON flow cell Mk I R9 version (FLO-MIN106). For bioinformatic analysis, default parameters were used for all software unless otherwise specified. Long-read demultiplexing was performed using Porechop (https://github.com/rrwick/Porechop). For short reads, libraries were prepared with a Nextera XT library preparation kit (Illumina, Netherlands) and sequenced on NextSeq 500 (midoutput kit v2, 2 × 150 bp) and MiSeq (MiSeq v3 chemistry, 2 × 300 bp) instruments, respectively.

Using ONT, a total of 1,463,879 reads (average read length [ARL], 1,307 nucleotides [nt]; *N*_50_, 2,416 nt) were obtained for isolate F-1367. For Survcare220, a total of 1,064,560 reads (ARL, 970 nt; *N*_50_, 1,417 nt) were obtained. The total number of short reads was 7,221,934 (ARL, 132 nt) for F-137 and 1,168,524 (ARL, 272 nt) for Survcare220. Hybrid genome assembly was carried out by Unicycler v0.4.6 combining long reads and Trimmomatic v0.36 filtered short reads (5, 6). For F-1367, assembly resulted in only one closed contig of 4,750,456 bp with a GC content of 55.97%. In the case of Survcare220, two circularized contigs of 4,766,761 bp (chromosome, 55.08% GC content) and 151,140 bp (52.05% GC content) resulted. Use of pMLST v2.0 identified a 151,140-bp contig with the IncFIB/IncFIA replicons that was classified as a plasmid. The final (filtered Illumina short-read and ONT long-read) coverages for the genome of F-1367 and Survcare220 were estimated to be 281- and 124- fold, respectively.

Both isolates exhibited an average nucleotide identity of 98.8% to *E. bugandensis* EB-247^T^ (GenBank accession number NZ_LT992502), as determined by JSpecies v1.2.1 using the “blastall” option. The *in silico* DNA-DNA hybridization score (*is*DDH) determined by the GGDC v2.1 tool was 89%, thus identifying the isolates as *E. bugandensis*. Since the initial discovery of *E. bugandensis* in 2011, this is the first time that this species has been isolated from clinical samples outside Africa. We note that unambiguous assignment is currently possible only by using whole-genome sequencing, and there is a need for rapid detection methods, e.g., using MALDI-TOF. The Enterobacter cloacae Multilocus Sequence Typing scheme database (https://pubmlst.org/ecloacae/, accessed 20 March 2019; powered by BIGSdb v1.22.3), which is comprised of different *Enterobacter* species, assigned F-1367 and Survcare220 to sequence type 795 (ST795) and the novel ST1140, respectively.

As of May 2019, only one other complete genome sequence of *E. bugandensis* is available in public databases. The complete genome sequences of these two new isolates will facilitate comparative genomic studies to identify genomic variations and describe the diversity of the pangenome of this highly pathogenic species.

### Data availability.

This whole-genome shotgun project has been deposited in DDBJ/ENA/GenBank under the accession number PRJNA531146. For *E. bugandensis* F-1367, Survcare220, and pSurvcare220, the accession numbers are CP039452, CP039453, and CP039454, respectively, and the versions described in this paper are the first versions. Sequence read data for the whole genome of *E. bugandensis* F-1367 and Survcare220 have been deposited in the Sequence Read Archive under the accession numbers SRR8858675 and SRR8858676, respectively.

## References

[1] Mshana SE, Gerwing L, Minde M, Hain T, Domann E, Lyamuya E, Chakraborty T, Imirzalioglu C 2011 Outbreak of a novel *Enterobacter* sp. carrying *bla*_CTX-M-15_ in a neonatal unit of a tertiary care hospital in Tanzania. Int J Antimicrob Agents 38:265–269. doi:10.1016/j.ijantimicag.2011.05.009. 21752606

[2] Jean S-S, Hsueh P-R 2017 Distribution of ESBLs, AmpC β-lactamases and carbapenemases among *Enterobacteriaceae* isolates causing intra-abdominal and urinary tract infections in the Asia-Pacific region during 2008–14: results from the Study for Monitoring Antimicrobial Resistance Trends (SMART). J Antimicrob Chemother 72:166–171. doi:10.1093/jac/dkw398. 27703058

[3] Pati NB, Doijad SP, Schultze T, Mannala GK, Yao Y, Jaiswal S, Ryan D, Suar M, Gwozdzinski K, Bunk B, Mraheil MA, Marahiel MA, Hegemann JD, Spröer C, Goesmann A, Falgenhauer L, Hain T, Imirzalioglu C, Mshana SE, Overmann J, Chakraborty T 2018 *Enterobacter bugandensis*: a novel enterobacterial species associated with severe clinical infection. Sci Rep 8:5392. doi:10.1038/s41598-018-23069-z. 29599516 PMC5876403

[4] Singh NK, Bezdan D, Checinska Sielaff A, Wheeler K, Mason CE, Venkateswaran K 2018 Multi-drug resistant *Enterobacter bugandensis* species isolated from the International Space Station and comparative genomic analyses with human pathogenic strains. BMC Microbiol 18:175. doi:10.1186/s12866-018-1325-2. 30466389 PMC6251167

[5] Bolger AM, Lohse M, Usadel B 2014 Trimmomatic: a flexible trimmer for Illumina sequence data. Bioinformatics 30:2114–2120. doi:10.1093/bioinformatics/btu170. 24695404 PMC4103590

[6] Wick RR, Judd LM, Gorrie CL, Holt KE 2017 Unicycler: resolving bacterial genome assemblies from short and long sequencing reads. PLoS Comput Biol 13:e1005595. doi:10.1371/journal.pcbi.1005595. 28594827 PMC5481147

